# Information processing in the LGN: a comparison of neural codes and cell types

**DOI:** 10.1007/s00422-019-00801-0

**Published:** 2019-06-26

**Authors:** Agnieszka Pregowska, Alex Casti, Ehud Kaplan, Eligiusz Wajnryb, Janusz Szczepanski

**Affiliations:** 1 Institute of Fundamental Technological Research, Polish Academy of Sciences, Pawinskiego 5B, 02–106 Warsaw, Poland; 2 Department of Mathematics, Gildart-Haase School of Computer Sciences and Engineering, Fairleigh Dickinson University, Teaneck, NY 07666 USA; 3 Icahn School of Medicine at Mount Sinai, New York, NY 10029 USA; 4 National Institute of Mental Health (NUDZ), Topolova 748, 250 67 Klecany, Czech Republic; 5 Department of Philosophy of Science, Charles University, Prague, Czech Republic

**Keywords:** Shannon information theory, Cat LGN, ON–OFF cells, Neural coding, Entropy, Firing rate

## Abstract

To understand how anatomy and physiology allow an organism to perform its function, it is important to know how information that is transmitted by spikes in the brain is received and encoded. A natural question is whether the spike rate alone encodes the information about a stimulus (*rate code*), or additional information is contained in the temporal pattern of the spikes (*temporal code*). Here we address this question using data from the cat Lateral Geniculate Nucleus (LGN), which is the visual portion of the thalamus, through which visual information from the retina is communicated to the visual cortex. We analyzed the responses of LGN neurons to spatially homogeneous spots of various sizes with temporally random luminance modulation. We compared the Firing Rate with the Shannon Information Transmission Rate , which quantifies the information contained in the temporal relationships between spikes. We found that the behavior of these two rates can differ quantitatively. This suggests that the energy used for spiking does not translate directly into the information to be transmitted. We also compared Firing Rates with Information Rates for X-ON and X-OFF cells. We found that, for X-ON cells the Firing Rate and Information Rate often behave in a completely different way, while for X-OFF cells these rates are much more highly correlated. Our results suggest that for X-ON cells a more efficient *“temporal code”* is employed, while for X-OFF cells a straightforward *“rate code”* is used, which is more reliable and is correlated with energy consumption.

## Introduction

A major challenge for modern neuroscience is to gain some insight into the mechanisms that underlie information processing in the brain, where spiking neurons communicate via discrete signals, called action potentials or spikes (Rieke et al. 1997). Since spiking activity is energy-intensive, energy use is associated with the efficiency of information transfer (Levy and Baxter 1996, 2002; Laughlin et al. 1998; Harris et al. 2015). A natural question then is how efficient are the various spiking schemes employed by neurons. Attwell and Laughlin (2001) analyzed the metabolic cost of different components of excitatory signaling and suggested that signaling-related energy consumption increases linearly with spiking frequency. More recent detailed models and experimental results [for example, Harris et al. (2015)] extend these calculations into the nonlinear regime. Harris et al. (2015), based on data from retinal ganglion cells (RGC) and thalamic synapses, fitted the relationship between firing frequency and information rate using exponential functions, although at low firing rate the relationship can be fit well with a linear function. Clearly, neuronal codes have a complex relationship with energy demands.

The structure and mechanisms of neuronal codes remain an open question (van Hemmen and Sejnowski 2006). Two main views, not mutually exclusive, have been discussed in the literature [for example, Shadlen and Newsome (1998)]. The first is based on the idea of a “rate code”, and it views the neural code as embedded in the spike frequency, while the second view, that of a “temporal code”, focuses on the temporal structure of spike trains. While the “temporal code” can be viewed as an advanced code, the “rate code” is directly linked to energy consumption, and depends linearly on spiking frequency (at least for low firing rates). To study the “temporal code,” we make use of Shannon’s information theory, using entropy rate estimators to quantify output information. It is known that an approximation of the entropy rate requires taking into account the temporal structure of the spike trains (Lempel and Ziv 1976; Amigo et al. 2004; Szczepanski et al. 2004; Gao et al. 2008; Crumiller et al. 2013; Pregowska et al. 2016; Bossomaier et al. 2016).

Modern attempts to quantify information transmission in the nervous system concentrate on treating neural communication processes in the spirit of Shannon’s Information Theory, with neuronal networks treated as communication channels (Shannon 1948; Cover and Thomas 1991; Rieke et al. 1997), along which spike trains transmit information. Since information is formally a difference of entropies (Bialek et al. 1991; Borst and Theunissen 1999; Nemenman et al. 2004; Paprocki and Szczepanski 2013a; Pregowska et al. 2015), entropy estimation has received a great deal of attention (Paninski 2003; Amigo et al. 2004; Kennel et al. 2005; Gao et al. 2008; Lesne et al. 2009). In this paper, we use the entropy rate estimator described by Strong et al. (1998), because of its high accuracy and low computational complexity, although other methods, which can be applied to large neuronal populations, have been advanced recently (Crumiller et al. 2011).

In this work, we study neuronal coding in the Lateral Geniculate Nucleus (LGN), which is the visual region of the thalamus. The LGN plays a key role in visual information transmission, as it is the main central connection from the optic nerve to the visual cortex. Hartline (1938) discovered that some visual neurons are excited by light onset (ON cells), while others are excited by light offset (OFF cells). Kuffler (1953) later showed that ganglion cells have concentric receptive fields, with an ON-center and an OFF surround, or *vice versa*. The signal paths generated by ON and OFF retinal ganglion cells continue in the LGN and converge on the same cortical cells (Schiller 1992). Both types of cells have similar dynamics, but they differ in several other physiological properties beyond a simple sign-inversion (Kaplan et al. 1987; Benardete and Kaplan 1999; Chichilnisky and Kalmar 2002; Zaghloul et al. 2003). The question of how stimulus contrast affects the communication of visual signals between retinal ganglion cells and LGN cells has been investigated by several groups. It was shown that increased stimulus contrast improves transmission from retina to LGN (Kaplan et al. 1987), and that LGN neurons exhibit greater contrast-dependent phase advance than their retinal inputs (Rathbun et al. 2016). Recently, there have been additional comparisons between the responses of ON and OFF cells in the mammalian visual system (Im and Fried 2015; Carandini 2016), but none has examined these differences from the perspective of information processing.

In this study , we investigate the role of the antagonistic surround of the receptive field on information processing and *Firing Rates* in X-ON and X-OFF LGN cells. We recorded spike trains from individual neurons in the cat LGN while presenting visual stimuli that consisted of spatially homogeneous, temporally noisy flashing spots across a range of sizes, which engaged more or less of the circuitry that produces the surround of the receptive field. Eleven cells have been analyzed in detail in this paper: 5 X-OFF and 6 X-ON cells. They were recorded in the LGN and were stimulated by the same spot sizes. Stimulus-dependent information was quantified directly from the entropy rates of these neural responses (Uglesich et al. 2009). Using the high-quality entropy estimators (Strong et al. 1998; Gao et al. 2008), we show that for small spots the information rate was clearly higher for X-ON cells than for X-OFF cells, while for larger spots the information transmission efficiencies were similar for the two cell types. Moreover, our results suggest that for X-ON cells a more efficient *“temporal code”* is employed, while for X-OFF cells a straightforward *“rate code”* is used.

The paper is organized as follows. In Sect. 2, the basic concepts of Information Theory and spike train characteristics are briefly reviewed. In Sect. 2.6, details concerning the experiments are described. The results of Information and Firing Rates comparisons are presented in Sect. 3. Section 4 contains a brief discussion and some concluding remarks.

## Methods and materials

After Adrian’s experiments (Adrian 1926) established that sensory neurons produce action potentials (spikes), it has been generally accepted that a spiking neuron communicates information through sequences of spikes called spike trains (Bialek et al. 1991; Nemenman et al. 2004). The entropy of spike trains was first estimated by MacKay and McCulloch (1952), which was probably the first application of Information Theory to the nervous system. Importantly, they viewed the spike train as being observed with some limited time resolution, which affects the transmission quality.

### Information processing and encoding

Spikes are a convenient way to transmit analog output variables over long distances, and much effort has been spent recently to analyze neuronal coding of spike sequences, especially its efficiency and the mechanisms and rules governing it (Rieke et al. 1997; Fairhall et al. 2001; Rokem et al. 2006; van Hemmen and Sejnowski 2006; Rolls 2007; London et al. 2008; Paprocki and Szczepanski 2011, 2013a, b; Urner et al. 2013; Lengler and Steger 2017; Voronenko and Lindner 2018). The problem of coding and decoding spike trains is central both to understanding how neurons process information (Rieke et al. 1997), and to neural prosthetic engineering, which links devices directly with the brain (Donoghue 2002). “Decoding” refers to the issue of how to “read out” the information contained in a set of neural spike trains. Hereafter we will consider the most popular and natural binary coding (Rieke et al. 1997; Pillow et al. 2011). This treatment is physically justified, since spike trains are recorded with a limited time resolution $\varDelta \tau$, so that in each time slice (bin) a spike is either present or absent. If we think of a spike in a bin as representing a “1” and no spike as representing a “0”, then, if we look at some time interval of length *T*, each possible spike train is equivalent to $\frac{T}{\varDelta \tau }=L$ binary digits. Such a sequence of bits $z^{L}$ can then be treated as an output of a stochastic process. To study the information rate over time, we apply a moving window approach. The information rate at the moment of time *t* is obtained by using a specific entropy rate estimator applied just to the signal data in the time interval $[t,t+T]$. In the selection of window length *T*, two conflicting constraints are important. The first is that *T* must be long enough to estimate the entropy rate with good accuracy (this depends on the estimation method used). The second requires the window *T* to be short enough to guarantee the local stationarity of the signal. This trade-off has to be checked on a case-by-case basis. Given the window and the encoding frequency $f=\frac{1}{\varDelta \tau }$, a spike train is represented at time *t* by a bit sequence of length *fT*. In order to obtain the *Information Rate* conveyed by the neurons, the encoding frequency *f* must also be chosen appropriately. In Szczepanski et al. (2003), we analyzed the influence of the number of bins used for encoding on the Information Transmission Rate (ITR), using responses of neurons of the primary visual cortex to visual stimulation (sinusoidal drifting gratings). We observed that starting from an encoding parameter of about 80 bin/s, the ITR saturates, i.e., increasing the number of bins did not significantly change the ITR. Therefore, in this article, a bin width of $1/80=$ 12.5 ms was adapted as the coding parameter. Moreover, our numerical calculations (Szczepanski et al. 2004) show that $T=5$ s represents a good trade-off between the two conflicting requirements mentioned above, and our analysis of other experimental data corroborates this choice (Amigo et al. 2004).

### Firing rate estimation

High level of variability and irregularity are typical of in vivo recordings of brain activity. Even when the conditions of the experiment are *exactly* repeated, the same neuron may generate quite different outputs from trial to trial. This variability can be caused by randomness in spike generation, and dissimilarity in neuronal processing during each trial. In classical physiology, the Firing Rate depends on the intensity of the stimulus. In general, the Firing Rate increases with increasing stimulus strength. Thus, understanding the relation between Firing and Information Rate is of high importance (Rieke et al. 1997; Churchland et al. 2010). The commonly applied Firing Rate definition involves the temporal average (Gerstner et al. 2014) and is given by:

1 $$\begin{aligned} F_\mathrm{R}=\frac{n_{_{\text {T}}}}{T} \end{aligned}$$

where $n_{_{\text {T}}}$ denotes spike count and *T* is the length of the time window. In practice, in order to get sensible averages, some reasonable number of spikes should occur within the time window. Since the messages are treated as outputs of a locally stationary stochastic process, the Firing Rate as defined by (1) is specific for a given information source, provided *T* is large enough. Thus, $n_{_{\text {T}}} \cdot \varDelta \tau$ can be related to the probability *p* of spike appearance, where $\varDelta \tau$ is the time resolution or bin size.

### Entropy and information in the Shannon sense

From a mathematical point of view, the information contained in a given spike train, after the encoding (binning) process, is represented as an output of a stochastic process, being in fact a sequence of symbols. It is assumed that the set of symbols (alphabet) is finite, and that the stochastic process representing the spike train has a stationary distribution (Ash 1965; Cover and Thomas 1991). Consider first a general case, in which *X* is a discrete random variable with probability function $p(x_{i} ),i=1,2,\ldots ,n$. Then, according to Shannon, the information transmitted by the event $X=x_{i}$ is equal to $-\log _{2}p (x_{i})$. In this sense, less probable events carry more information. The average information transmitted by all realizations of *X* is called entropy and is given by:

2 $$\begin{aligned} H(X):=-\sum _{i=1}^{n} p(x_{i}) \log _{2} p(x_{i}) \ . \end{aligned}$$

Now, let $Z^{L}$ be the set of all words (i.e., blocks, for example binned spike trains) of length *L*, built of symbols (letters) from the finite alphabet $Z=\{z_{1}, z_{2},\ldots ,z_{m}\}$. Each word $z^{L}$ can be treated as a message carried by the corresponding spike train. If $P(z^{L})$ denotes the probability that the word $z^{L} \in Z^{L}$ occurs, then the information carried by this word, according to Shannon’s Theory, is equal to

3 $$\begin{aligned} I(z^{L}):=-\log _{2} P(z^{L}) \ . \end{aligned}$$

Thus, the expected or average information, called Shannon block entropy, of random variable $\{Z^L\}$ corresponding to the words of length *L* according to (2) is

4 $$\begin{aligned} H(Z^{L})=- \sum _{z^{L} \in Z^{L}} P(z^{L}) \log _{2} P(z^{L}) \ , \end{aligned}$$

The entropy of spike trains quantifies how much information these spikes could provide. The adequate measure for estimation of efficiency of an information source is the information transmitted, on average, by a single symbol. This measure is called *Entropy Rate* or *Information Transmission Rate* , and is defined (Ash 1965) as:

5 $$\begin{aligned} \hbox {ITR}(\{Z\})=\lim _{L \rightarrow \infty } \frac{1}{L} H(Z^{L}) \ . \end{aligned}$$

Entropy rate is an important invariant for ergodic stochastic processes (Cover and Thomas 1991) and is often applied in the analysis of biological signals. Based on the equation $H(X,Y)=H(X)+H(Y|X)=H(Y)+H(X|Y)$, which is valid for any random variables *X*, *Y*, it was proven in (Ash 1965) that for any Information Source Eq. (5) can be applied to estimate the Mutual Information (6) between input and output signals.

The fundamental concept of Shannon‘s Theory (Shannon 1948) is Mutual Information MI(*X*, *Y*), which quantifies the information dependence of random variables or stochastic processes. The MI(*X*, *Y*) between input and output signals is defined as

6 $$\begin{aligned} \begin{aligned} \hbox {MI}(X,Y)&{=} H(X){-}H(X|Y)=H(X)+H(Y)-H(X,Y)\\&=H(Y)-[H(X,Y)-H(X)]\ , \end{aligned} \end{aligned}$$

where *H*(*X*|*Y*) is a conditional entropy of *X* under the assumption *Y* is known and *H*(*X*, *Y*) is the joint entropy of *X* and *Y*. Since $H(X,Y) - H(X)>0$, by equation (6) the *H*(*Y*) is, in fact, the upper bound of MI(*X*, *Y*), and it is closely related to the capacity (being maximal Mutual Information over input probability distributions) of the communication channel. Capacity is the most important quantity describing a communication channel because it characterizes possibilities of decoding with assumed error. Clearly, *H*(*Y*) itself also depends on the input signal *X*, i.e., for two different signals $X_{1}$ and $X_{2}$ the output entropies $H(Y_{1})$ and $H(Y_{2})$ will be, in general, also different. Thus, measuring *H*(*Y*) we have information about the behavior of MI(*X*, *Y*).

### Entropy rate estimators

Over the past two decades, entropy estimation has received a lot of attention (Paninski 2003; Amigo et al. 2004; Kennel et al. 2005; Gao et al. 2008; Lesne et al. 2009; Crumiller et al. 2011). Several entropy rate estimators have been proposed (Gao et al. 2008). The basic requirements of these estimators are, at least, local stationarity and ergodicity of the underlying stochastic process. In Szczepanski et al. (2003) and Amigo et al. (2004), we have used the Lempel–Ziv Complexity (LZC) (1976). These estimators are known to be consistent only under certain restrictive conditions on the data, i.e., to converge quickly enough to the entropy value. Here, we apply the estimator of Strong et al. (1998) due to its low computational complexity and high accuracy. This approach, commonly called the *Direct Method*, is based on calculating block entropies using observed frequencies of words $z^{L}$ for a few consecutive and relatively small lengths *L*. Then a line *h*, which best fits the points $(\frac{1}{L}, \frac{H(Z^{L})}{L})$, is determined. Finally, with $\frac{1}{L} \rightarrow 0$, *h* is extrapolated to the point of $(0, H(\{Z\}))$. Using this method, we are able to get fast and reliable entropy rate estimations. Our simulations for Markov processes confirmed the high quality of this method (Paprocki and Szczepanski 2013a; Szczepanski et al. 2004), while the quality of Lempel–Ziv estimator was verified in Szczepanski et al. (2003) and Amigo et al. (2004). In these papers, we presented simulations showing the convergence rate of entropy estimators we used as a function of word length. It turned out that the estimation error was relatively low (about 4% for the sequences of 400 bits).

**Fig. 1:**
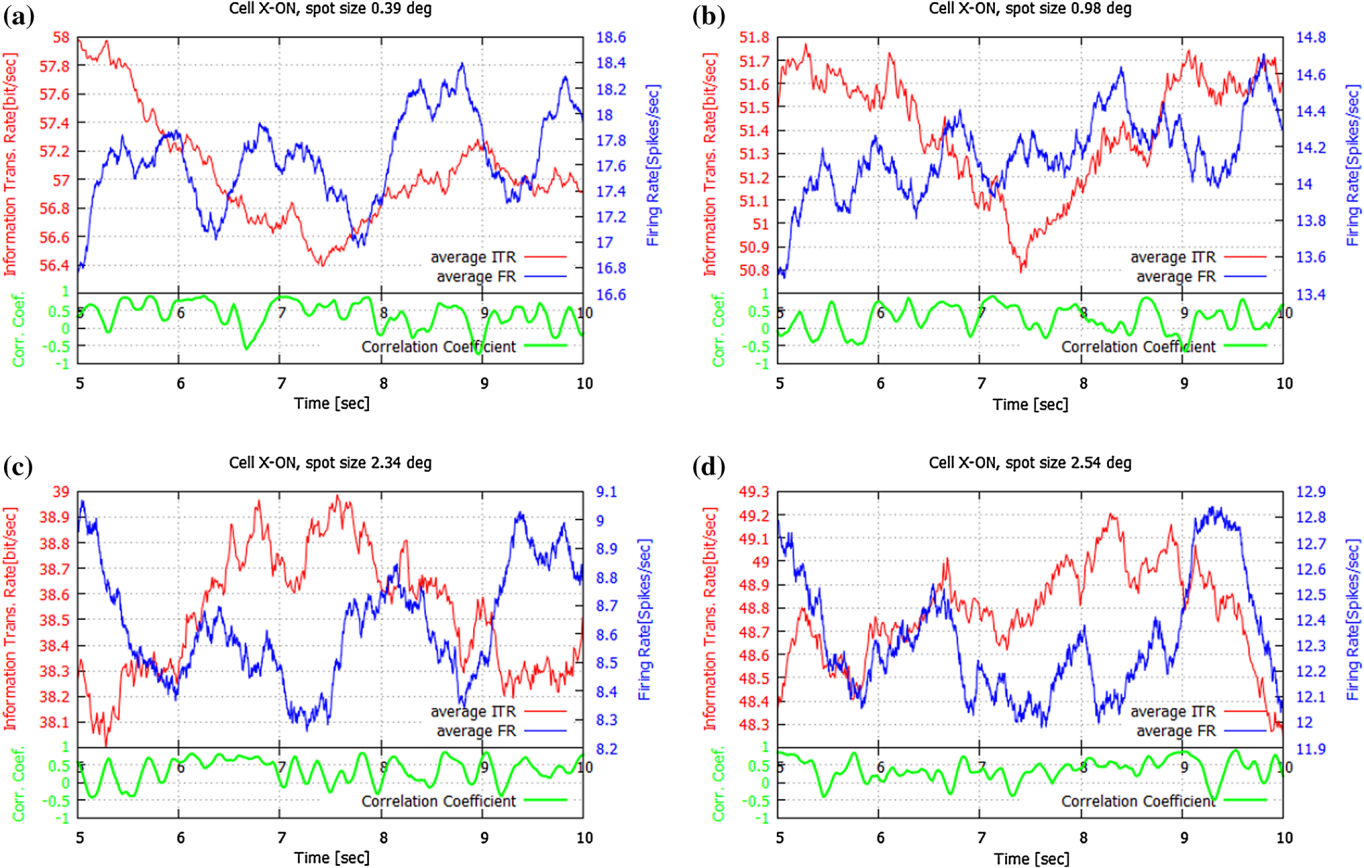
Information Transmission Rate and Firing Rate for one cell (X-ON1) for four different spot sizes **a**$0.39^{\circ }$; **b**$0.98^{\circ }$; **c**$2.34^{\circ }$; **d**$2.54^{\circ }$. Here, the center size was approximately $1.176^{\circ }$. Only the averages are shown, since the standard deviation values are relatively small compared with the averages (0.8%), and the deviation curves largely overlap with the curves for the averages. At the bottom of each panel, the PCC between ITR and $F_\mathrm{R}$ is also shown (in green). Note that there are periods where the ITR decreased while at the same moment $F_\mathrm{R}$ decreased and *vice versa*. This is confirmed by PPC values, which mostly oscillate around zero. For details see Sect. 3 (colour figure online)

**Fig. 2:**
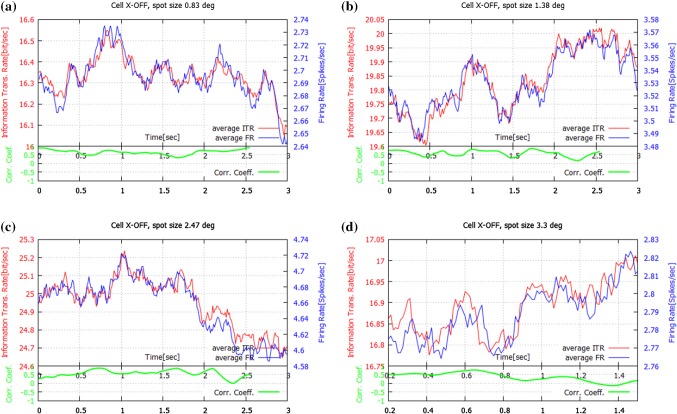
Information Transmission Rate and Firing Rate for one cell (X-OFF2) for four different spot sizes **a**$0.83^{\circ }$; **b**$1.38^{\circ }$; **c**$2.47^{\circ }$; **d**$3.3^{\circ }$. Here, the center size was approximately $1.1^{\circ }$. Only the averages are shown, since the standard deviation values are relatively small compared with the averages (1.5%), and the deviation curves largely overlap with the curves for the averages. At the bottom of each panel the PCC between ITR and $F_\mathrm{R}$ is also shown (in green). Observe, that ITR and $F_\mathrm{R}$ have very similar time courses. For details see Sect. 3 (colour figure online)

**Fig. 3:**
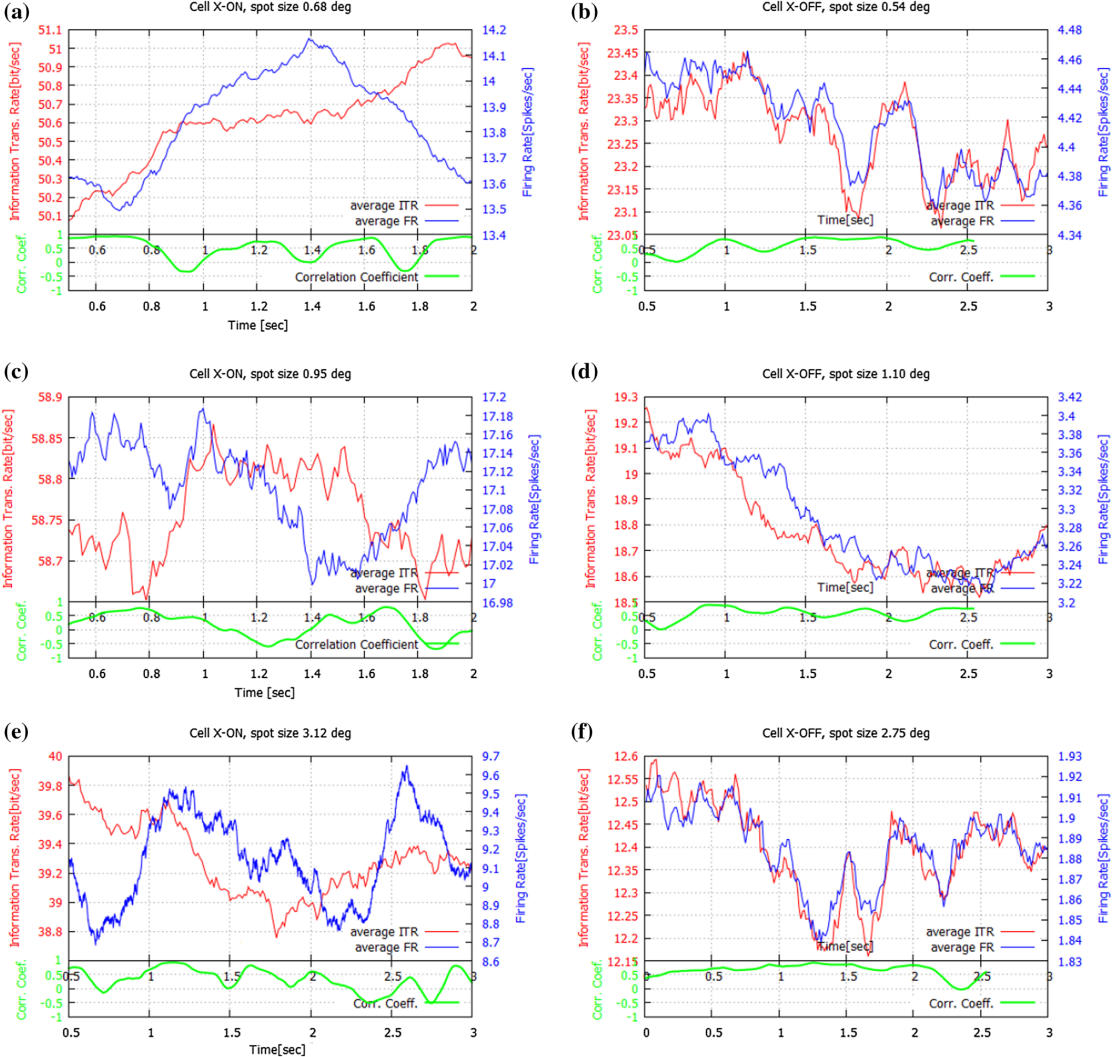
Comparison of ITR and $F_\mathrm{R}$ behavior for the same period of time for four LGN cells: two ON (left column) and two OFF (right column), at several spot sizes: **a** X-ON5, $0.68^{\circ }$; **b** X-OFF3, $0.54^{\circ }$; **c** X-ON5, $0.95^{\circ }$; **d** X-OFF3 $1.10^{\circ }$, **e** X-ON1, $3.12^{\circ }$; **f** X-OFF1, $2.75^{\circ }$. Only the averages are shown, since the standard deviation values are relatively small compared with the averages (0.8–1.5%), and the deviation curves largely overlap with the curves for the averages. For the X-ON cells, the courses of ITR and $F_\mathrm{R}$ are qualitatively different, while for X-OFF cells these quantities follow a very similar time course. Under each panel, the corresponding PCC between ITR and $F_\mathrm{R}$ curves are shown (in green) (colour figure online)

### Relation between firing rate and entropy rate

As mentioned in Sect. 1, an important question when studying information processing in the context of brain physiology is the relationship between Information Transmission and Firing Rates. It is known that Information Transmission Rate is sensitive to the variability of the encoded spike train (Szczepanski et al. 2004; Lempel and Ziv 1976; Paprocki and Szczepanski 2011, 2013a, b). The way of generating spike trains is usually modeled by (homogeneous or non-homogeneous) Poisson or Markov stochastic processes (Bialek et al. 1991; van Hemmen and Sejnowski 2006; Brown et al. 2002). For such processes, there is no straightforward correlation between firing rate and information rate. For example, for an Information Source which is governed by a Markov process with high transition probabilities from the state “no spike” to the state “there is spike” and *vice versa* (e.g., above 0.9), typical sequences generated are almost periodic, like $010101 \dots$ . Thus, in such cases the firing rate is high (since the probability of spike in a bin is close to 0.5), while the information carried by such almost periodic sequences is close to 0. Therefore, our approach concerning the interpretation of coding strategy is based primarily on the analysis of the Pearson Correlation Coefficient (PCC) between Information Transmission Rate and Firing Rate. It is known that PCC does not depend on the inner properties of the random variables. It depends only on the joint probabilities of these random variables and compares their variability.

### Experiments

The experimental methods we used were similar to those previously described in (Kaplan and Shapley 1984; Ozaki and Kaplan 2006; Casti et al. 2008; Uglesich et al. 2009). They conformed to the requirements and regulations of the NIH, and were approved by the Mount Sinai IACUC. Nine adult cats were anesthetized initially with an intramuscular injection of xylazine (Rompun, 2 mg/kg) followed by ketamine hydrochloride (Ketaset, 10 mg/kg), and then given propofol (diprivan) as needed during surgery. The animal was mounted in a stereotaxic apparatus and phenylephrine hydrochloride $(10\%)$ and atropine sulfate $(1\%)$ were applied to its eyes. The animal’s heart rate and blood pressure monitored the depth of anesthesia. Signs of distress, such as salivation or increased heart rate, were watched for, and the infusion rate of the anesthetic was adjusted as necessary. The eyes were refracted, and correcting lenses focused the eyes for viewing distances of 57–114 cm. The tip of the recording electrode was brought close to the cell body of an LGN neuron, to ensure a clean and stable extracellular recording of both the LGN spike and its retinal input (Bishop 1953; Kaplan and Shapley 1984). In this paper, we analyze only the LGN spikes.

Once a well-isolated LGN cell was identified, its spatiotemporal receptive field was mapped using a $16\times 16$ checkerboard, in which each check was modulated by an m-sequence (Reid et al. 1997), presented on a black and white CRT (frame rate: 160 Hz), with which we were able to find the size and location of the receptive field center. We further characterized the cell as ON or OFF and X or Y, using contrast-reversing gratings or a small spot flashing at an optimal temporal frequency.

For the Information and Firing Rate calculations, we used a visual stimulus that consisted of a spatially homogeneous, temporally noisy flashing spot centered on the cell’s receptive field. The temporal pattern of this stimulus followed a “natural” luminance time series, as described by van Hateren (1997), but without preserving the temporal correlations. A stimulus set at a given spot size typically consisted of 256 trials, each 8 s long, for which 128 *repeat* (R) segments of the same stimulus sample were interleaved with 128 *unique* (U) segments, in the pattern RU RU ... RU. During each run, the spot size was fixed, and only the luminance level was varied. For some recordings, just 32 unique trials were recorded, with 4 repeat segments between the unique segments in the pattern RRRRU RRRRU ... RRRRU.

In total we analyzed here 5 X-OFF and 6 X-ON cells, recorded in 9 cats. The cells analyzed in this work are a sub-sample of a larger collection of cells recorded from these cats. Most of the neurons in this larger sample were not recorded for a sufficiently long time to allow the presentation of several spot sizes, which was required for our analysis. Although the number of cells is small, it is comparable to numbers used in other similar studies, such as Reinagel and Reid (2000). For three of the X-OFF cells the stimulus presentation order was RU RU RU, and for two other X-OFF cells it was RRRRU RRRRU ... RRRRU. For four of the X-ON cells the stimulus sequence was RU RU ... RU, and for the remaining two X-ON cells the stimulus was RRRRU RRRRU ... RRRRU.

## Results

We analyzed the relation between ITR, the transmission rate of information (in Shannon sense, Eq. 3) and the corresponding Firing Rate $(F_\mathrm{R})$, addressing two questions:

- How does the cell type (ON or OFF) affect this relation?
- How does the size of the stimulus affect the relation between ITR and $F_\mathrm{R}$?

To answer the first question, we compared signals recorded from X-ON and X-OFF LGN cells. To address the second question, we analyzed the time evolution of ITR and $F_\mathrm{R}$ across a range of increasing spot sizes that gradually engaged most or all of the cell’s receptive field surround. In any given set of repeat (*R*) and unique (U) stimulus trials, the spot size was kept fixed. As it is presented in Section Information Processing and Encoding, at each moment of time *t* we estimated the Information Rates and Firing Rates from a 5 s time window moving over the entire duration of the experiment. We binned spike trains as has been commonly done in the literature (Rieke et al. 1997).

For X-OFF cells, we estimated ITR and $F_\mathrm{R}$ within 8 s *R* segments in three experiments, and 32 s RRRR segments in two other experiments (see details of experiment in Sect. 2.6), while for X-ON cells we estimated these quantities in two experiments for 32-s intervals (RRRR), and in two other experiments for 8-s intervals (R). For cells with the stimuli-order RU the time interval of estimation is 3 s long, i.e., duration of R (8 s) minus duration of the moving window ($T=5$ s). We repeated these calculations 128 times, once for each repeat trial *R*. Next, for each moment of time *t* we calculated the average and standard deviation over these 128 estimation results. We performed similar calculations for cells with the stimuli-order RRRR. In this case, the time interval of estimation was for one experiment also 3 s, and for another experiment it was 27 s long (32–5 s = duration of RRRR-*T*).

For both OFF and ON cells, the *standard deviation* values were relatively small compared with the average values (0.8–1.5%). Thus, since the deviation curves largely overlap the curves for the averages, for clarity we show only the average values in Figs. 1, 2, and 3.

### Cell type (ON or OFF) influence on ITR and $F_\mathrm{R}$

In this section, we present results comparing the Information Transmission Rate with Firing Rate for ON and OFF cells for various stimulus spot sizes. The spot sizes ranged from 0.39$^{\circ }$ to 28.00$^{\circ }$ of visual angle (for details see Sect. 2.6). The visual stimulus parameters were chosen to address the role of the antagonistic receptive field surround. First, a small spot was shown on the screen, and then, in the next experiments, the spot size was successively increased so that the screen was finally completely filled.

Our calculations show that for the ON cells under considerations there were periods of time for which the ITR and $F_\mathrm{R}$ curves behaved quite differently. For all spot sizes, there are periods where the Transmission Rates increased while at the same moment the Firing Rates decreased and *vice versa*. Typical cases are presented in Figs. 1 and 3 (left column).

Figure 1 shows the ITR and $F_\mathrm{R}$ for the X-ON cell for several spot sizes, (a) small (< 1.0$^{\circ }$), (b) medium (1.0 $^{\circ }$–2.0$^{\circ }$) and (c, d) large (> 2.0$^{\circ }$). The divergences between ITR and $F_\mathrm{R}$ are especially visible in Fig. 1 panel (a) 5.0–6.0 s and 6.5–6.8 s, panel (b) 5.0–6.8 s, panel (c) 5.2–5.5 s and 6.2–6.9 s, and panel (d) 5.0–5.5 s. In Fig. 3 these divergences are clear in panel (a) 1.7–2.0 s, panel (c) 1.7–2.0 s and panel (e) 1.5–2.0 s and 2.5–2.8 s.

In contrast, in Figs. 2 and 3 (right column) for OFF cells we observed a completely different relationship between ITR and $F_\mathrm{R}$. Namely, the two measures have very similar time courses. This can be seen during the entire time: an increase in the number of spikes leads to a higher Transmission Rate, while a decrease of this number causes a decrease in the Transmission Rate.

The divergences between ITR and $F_\mathrm{R}$ are reflected in the corresponding Pearson Correlation Coefficients, which are also shown in Figs 1, 2 and 3 (green trace). The time course of the PCC was estimated over a moving time window of 0.25 s duration. The *PCC* values for X-OFF cells are clearly high for small and medium spots, while for X-ON cells they are about two times lower. Table 1 presents the PCCs between ITR and $F_\mathrm{R}$ for the ON and OFF cell types for different spot sizes.

In Table 1, we see that for small, medium and large spot sizes the correlations are lower for X-ON cells, and |PCC| is not greater than 0.4, while for X-OFF cells we have higher correlations, and |PCC| is in the range of 0.5–0.7. An interesting observation is that for X-OFF cells stimulated with very large spots, i.e., ones that stimulated both center and surround of the receptive field, we also have higher correlations: the value of |*PCC*| is equal to 0.6.

### Stimulus size influence on information and firing rates

We now address the second question listed above: how does stimulus size affect the behavior of ITR and $F_\mathrm{R}$ for both cell types (ON or OFF) in the LGN, in other words, what role does the antagonistic surround play in the information transfer? In Fig. 3 we show results of a comparison of ITR and $F_\mathrm{R}$ for these two cell types for small (< 1.0$^{\circ }$), medium (1.0$^{\circ }$–2.0$^{\circ }$), and large (> 2.0$^{\circ }$) spot sizes. We observed that ON cells transmit more information than OFF cells, particularly for smaller spot sizes, where only the receptive field center is stimulated. In this case, the loss of information by OFF cells when compared to ON cells is $70\%$ for the cells presented in Fig. 3, all panels. Moreover, for small spots the Firing Rates for ON and OFF neurons can also differ significantly (Fig. 3a). X-ON neurons can fire three times more often than X-OFF cells (Fig. 3, all panels). Moreover, for X-OFF cells the ITR and $F_\mathrm{R}$ time courses, in corresponding time intervals, are much more correlated then they are for X-ON cells. This is especially visible in Fig. 3b, d, f.

**Table 1:** Pearson correlation coefficients between ITR and $F_\mathrm{R}$ for the two cell types (ON, OFF) and three spot sizes

	Small spot size	Medium spot size	Large spot size
	< 1.0$^{\circ }$	$1.0^{\circ }$–$2.0^{\circ }$	> 2.0$^{\circ }$
X-ON	0.35–0.40	0.30–0.45	0.30–0.35
*p* values	0.01	0.025	0.01
X-OFF	0.60-0.65	0.58–0.62	0.50–0.70
*p* values	0.025	0.075	0.025

Note that PCC is clearly higher for OFF cells

In Table 2, the ranges of values reached by ITR and $F_\mathrm{R}$ are shown as a function of the spot size, i.e., small (< 1.0$^{\circ }$), medium (1.0$^{\circ }$–2.0$^{\circ }$), and large (> 2.0$^{\circ }$) for X-ON and X-OFF cells. For small spot sizes in the case of X-ON cells ITR is in the range 50.0–58.0 [bit/s], while for X-OFF cells it is in the range of 16.0–23.0 [bit/s]. For medium stimulus sizes, the ITR values are also greater for X-ON cells (51.0–58.0 [bit/s]) than for X-OFF cells (18.5–20.0 [bit/s]). When we consider large spot sizes, the ITR values are in the range (38.0–49.0 [bit/s]) and (12.5–25.2 [bit/s]) for X-ON and X-OFF cells, respectively. Note that a given $F_\mathrm{R}$ leads to a higher ITR in X-ON cells than in X-OFF cells. These observations also support the hypothesis that while X-OFF cells use *rate coding*, the X-ON cells use the more sophisticated *temporal coding*.

## Discussion

In this paper, we have investigated two questions related to the processing of luminance information in the mammalian visual system, and its relation to the type of cell (ON or OFF), using methods from Shannon’s Information Theory. Specifically, we asked:

1. How does the cell type (ON or OFF) affect information processing? To our knowledge, ours is the first attempt to compare ON and OFF cells from the perspective of Shannon information.
2. What role does the antagonistic surround play in the transfer of information through the LGN? We compared Firing Rate, $F_\mathrm{R}$, with Information Transmission Rate, ITR, for spots of various sizes: small ones that stimulated only the receptive field center, and large ones that stimulated both center and surround.

**Table 2:** Spot size influence on information and firing rates

Spot size ($^{\circ }$)	ITR range (bit/s)		$F_\mathrm{R}$ range (spikes/s)	
	X-ON	X-OFF	X-ON	X-OFF
Small < 1.0$^{\circ }$	50.0–58.0	16.0–23.0	14.0–18.0	2.6–4.5
Medium 1.0$^{\circ }$–2.0$^{\circ }$	51.0–58.0	18.5–20.0	13.0–17.0	3.2–3.6
Large > 2.0$^{\circ }$	38.0–49.0	12.50–25.2	8.0–12.0	1.8–4.7

We found that $F_\mathrm{R}$ and ITR behaved rather differently (were poorly correlated) for ON- but not for OFF- cells, *as long as the stimulation was confined to the receptive field center.* Once the surround of the receptive field was stimulated as well, this difference disappeared. This result suggests that during center stimulation, which is typically the most effective and induces the highest firing rate, OFF cells use an energy-expensive *rate code* to transmit information, while ON cells employ a more sophisticated, energy-efficient *temporal code*. Overall, it appears that the two cell types are designed to transmit roughly the same amount of visual information. Since ON cells fire many more spikes, the information per spike is, necessarily, lower.

### Spike-time encoding

When analyzing neural encoding, the characteristics of the stimulus should be taken into account. Some relevant examples come from studies in which stimuli were presented only briefly (VanRullen et al. 2005; Gollisch and Meister 2008; Cerquera and Freund 2011). Such stimuli systematically influence the first-spike latency following a stimulus onset. Gollisch and Meister (2008) discovered that the first-spike latency allowed them to reconstruct the spatial structure of their stimuli, and argued that the response characteristics they observed resulted from the different kinetics of the ON and OFF pathways. Similarly, Cerquera and Freund (2011) found that the information derived from latencies of the first spikes (supplemented by the second and third spike times) allowed them to identify the velocities of moving light patterns projected onto the isolated turtle retina. Lestienne (2001), however, emphasized that millisecond spiking precision (an essential requirement for a temporal code) typically requires dynamic stimulation or sharp changes in the stimuli, a notion that was advanced previously by Mainen and Sejnowski (1995). We note that conveying information through the latency of the first spike is a form of a (minimalistic) temporal coding (Meister and Berry 1999; Van Rullen and Thorpe 2001; VanRullen et al. 2005).

Srinivasan et al. (1982) argued that to avoid redundancy and protect transmission against noise, there are interneurons that exploit the spatial mean values of signals rather than specific deviations from them. Since a chemical blockade of the ON pathway alters the receptive field, especially for stimuli that are brighter than the background, it seems that ON cells are mainly responsible for precise processing of mean values (Zaghloul et al. 2003). Srinivasan et al. (1982) suggested that the antagonistic surround of the receptive field plays a role in *Redundancy Reduction* (Barlow 1961), which allows the brain to conserve energy by communicating only non-redundant information. Our findings can be taken as support for that suggestion, although it is unclear why this should apply only to ON cells and not to OFF cells, given the essential similarity of their underlying retinal circuits (but see below). Perhaps this is due to the fact that, on average, under the ambient illumination commonly used in visual experiments, the firing rate of ON cells is $\sim 3$ times as high as that of OFF cells [see Table 1 in Kaplan et al. (1987) and Passaglia et al. (2001)], so their need to conserve energy is much more pressing. We note that Carandini (2016) has recently suggested that the difference between ON and OFF cells has to do with the fact that ON cells responses saturate at high intensities. Perhaps these cells must resort to energy-conserving temporal coding when their firing rate resources are depleted, while OFF cells, which fire at a much lower rate, can continue to use a more wasteful rate code.

### Firing rate

The threefold difference between the mean firing rates of ON and OFF cells in our data raises the possibility that the difference we observed between ON and OFF LGN cells is actually due to the *firing rate*, and not to the cell type *per se.* It is not obvious how lowering the firing rate of a cell (all other things being equal) would cause a change in the relationship between $F_\mathrm{R}$ and ITR, as quantified by the *PCC*, although we might expect a higher mean to lead to higher variability. To definitely establish the role of firing rate, one would have to compare ON cells with low firing rate with OFF cells of similar firing rates, but such a comparison was not possible with our data set.

### Firing precision

In addition to the mean firing rate, an important characteristic of the spike train is the *temporal precision* of the firing. Butts et al. (2007) have shown that the firing precision can change with the stimulus, to maintain a relatively constant relationship between the time scales of the stimulus and the response to it. The stimulus used in our experiments was similar to the one used by Reinagel and Reid (2000) and to one of those used by Butts et al. (2007), which typically elicits high precision firing. This precision has a bearing on the appropriate *encoding window* (Theunissen and Miller 1995), which is related to the length of the symbol in the neuron’s alphabet.

### Anatomical considerations

Several studies have described differences in the retinal circuitry that lead to the ON and OFF cell types (Zaghloul et al. 2003). In particular, Margolis and Detwiler (2007) point out that the OFF ganglion cells receive more amacrine input, and as a result manifest different patterns of firing at rest, which are not seen in cells of the ON pathways. It remains to be seen whether such anatomical differences could account for the functional differences uncovered by our analysis.

### Other ON/OFF differences

Recently, Poria and Dhingra (2015) reported that blocking glycine receptors with strychnine abolished oscillatory activity in OFF retinal ganglion cells (RGCs) in rd1 mouse retina, but there was no such change in ON RGCs. Jiang et al. (2015) compared ON and OFF cells in the parvocellular layers of the LGN of the awake, behaving macaque monkey. They report contrast sensitivity differences between the two cell types, and- surprisingly—a close relationship between the monkey’s perceptual performance and the responses of ON- but not OFF- cells. Although neurons recorded in the cat LGN are more similar in their response to contrast to the primate *magnocellular*, rather than *parvocellular* LGN cells [see Figure 5 in Shapley and Perry (1986) and Kaplan and Shapley (1986)], had worked on anaesthetized, rather than awake animals, our finding that LGN ON cells transmit more information to the visual cortex than do OFF cells could, perhaps, account for the findings of Jiang et al. (2015).

### Energy consideration

Recent studies (Harris et al. 2015) have shown that the properties of thalamic relay synapses are tuned to maximize bits of information transmitted per ATP molecule used, rather than bits of information transmitted per second. Our data consisted only of extracellularly recorded spike trains, and we had no direct access to the synaptic currents that underlie these spike trains. Thus we could not comment on the metabolic energy cost of the cellular processes that underlie the neural information processing we were studying. However, there is no reason to believe that ON and OFF thalamic neurons have different synaptic mechanisms. As mentioned above, the crucial difference between these populations seems to be the average firing frequency, which is due to the underlying retinal circuitry. We note, however, that the report in Harris et al. (2015) of increased information transmission across thalamic synapses with larger EPSCs could account for the observation made in Kaplan et al. (1987) of improved transmission from retina to LGN with increased stimulus contrast.

In conclusion, our results suggest a novel, intriguing difference between the information coding strategy of the ON and OFF cell populations, a difference that is designed to maximize energy efficiency in the transmission of visual information from the retina to the rest of the brain.
